# Mapping the Primary and Secondary Metabolomes of Carob (*Ceratonia siliqua* L.) Fruit and Its Postharvest Antioxidant Potential at Critical Stages of Ripening

**DOI:** 10.3390/antiox10010057

**Published:** 2021-01-05

**Authors:** Marios C. Kyriacou, Chrystalla Antoniou, Youssef Rouphael, Giulia Graziani, Angelos Kyratzis

**Affiliations:** 1 Department of Vegetable Crops, Agricultural Research Institute, 1516 Nicosia, Cyprus; chrystalla.antoniou@ari.gov.cy (C.A.); a.kyratzis@ari.gov.cy (A.K.); 2 Department of Agricultural Sciences, University of Naples Federico II, 80055 Portici, Italy; youssef.rouphael@unina.it; 3 Department of Pharmacy, University of Naples Federico II, 80131 Naples, Italy; giulia.graziani@unina.it

**Keywords:** antioxidant assays, catechins, condensed tannins, flavonoids, HPLC-RI, hydrolyzable tannins, ion chromatography, Orbitrap LC-MS/MS, sugars

## Abstract

Six critical stages corresponding to major morphophysiological events in carob fruit ripening were defined, and changes in the primary and secondary metabolome and in vitro antioxidant capacity were examined in two genotypes collected at low (15 m) and high (510 m) altitudes from genetically identified and georeferenced trees. Soluble carbohydrates were analyzed by HPLC-RI, macro-minerals by ion chromatography coupled to conductivity detection and polyphenols by UHPLC-Q-Orbitrap-HRMS. spectroscopy facilitated assays for condensed tannins and in vitro free-radical scavenging capacity of 1,1-diphenyl-2-picrylhydrazyl (DPPH) and ferric-reducing antioxidant power (FRAP). The fruit respiration rate and moisture content declined sharply during the transition from the breaker to green pedicel stage. Sugar accumulation spiked at the onset of fruit coloration and culminated at 498.7 ± 8.4 mg g^−1^ dry weight (dw) in the late ripe stage, while the ratio of reducing sugars to sucrose decreased from 3.45 ± 0.32 to 0.41 ± 0.02. The total phenolic compounds and condensed tannins declined with ripening, particularly during the transition from the breaker to green pedicel stage. Eighteen polyphenols were identified and quantitated, with catechins and hydrolyzable tannins being dominant until the onset of fruit coloration. The transition to the green pedicel stage signaled a precipitous decline (90.9%) in catechins, hydrolyzable tannins (60.2%) and flavonol glycosides (52.1%) concomitant to the rise in gallic acid, which was putatively fueled by the enzymatic hydrolysis of gallotannins in immature fruit. Catechins, hydrolyzable tannins and flavone glycosides were more abundant at higher altitudes and gallic acid at lower altitudes. An antioxidant capacity was also favored by higher elevations and declined with ripening, particularly after the breaker stage. Correlations with FRAP and DPPH assays were significant for the total phenolic content, condensed tannins, catechins and hydrolyzable tannins. The highest correlation factors were obtained for epigallocatechin-gallate (r = 0.920 and r = 0.900; *p* < 0.01). Although the sharp drop in hydrolyzable and nonhydrolyzable tannins and catechins compromised the in vitro antioxidant capacity at physiological maturity, it also reduced the astringency and configured a palatable organoleptic fruit profile. These changes unraveled significant episodes in the ripening-related secondary metabolism of the carob fruit. They further highlighted the value of immature carob as a potent source of gallotannins, with putative in vivo anti-inflammatory action, and of catechins beneficial in preventing and protecting against diseases caused by oxidative stress.

## 1. Introduction

The carob (*Ceratonia siliqua* L.) is a leguminous tree (Fabaceae) considered a minor or underutilized crop that is encountered in feral and selected forms throughout the Mediterranean basin. A renewed interest in carob fruit is driven by the crop’s resilience to abiotic stress exacerbated by climate change in the Mediterranean [1], as well as a growing demand for both carob pulp and seed by the food industry [2]. Carob pulp is primarily exploited for the high calorific value of its pulp that is rich in sugars (40–56%) and for its seed germ, which is the source of galactomannans that comprise the locust bean gum (LBG) used widely as a food stabilizer [3,4]. The sugar-dense pulp is a by-product of the kibbling process, but it is also rich in other bioactive components, such as polyphenols, cyclitols and soluble pectins [5,6]. These components have been ascribed wide-ranging biological properties potentially conferring significant health benefits toward the prevention and control of such diseases as colon cancer and hepatocellular carcinoma, diarrheal syndrome, hypercholesterolemia and diabetes [2,7,8]. Consequently, several carob pulp products (e.g., powder, fiber, juice and molasses) have become functional components of upcoming health-promoting food products [9,10].

The carob presents a unique phenological profile compared to other Mediterranean fruits as it blooms in late-summer to autumn (September to mid-November), and the fruit develops and ripens on the tree for an exceptionally long period of 10–11 months [1]. Fruit growth is sigmoidal, progressing slowly in the winter, followed by fast growth in the spring and slowing down in the summer before coming to a halt in June–August when, at ripening, dehydration and coloration of the pod take place [11]. The exact chronology of fruit ripening can be influenced putatively by various factors, including the growth environment (especially altitude), alternate bearing (“on” or “off” crop year) and genotype [1,12]. Natural pod abscission signals the end of the ripening process; however, the resistance to fruit drop differs between cultivars, with some having a high resistance to abscission (e.g., “Tylliria”, “Ramillete” and “Negra”), while others, low (e.g., “Rojal”, “Koundourka” and “Sfax” [3]). This is a trait that affects the efficiency of harvesting, performed manually at the end of summer to beginning of autumn by knocking the ripe pods using long poles. The commencement of harvesting operations is usually signaled by the readiness of kibbling plants to receive crop loads once the fruit is fully colored and its moisture content is reduced. A wide variation (8–18%), however, may be traced in the literature as the fruit moisture threshold for commencing harvesting [1,13]. Notwithstanding this variation that may reflect cultivar differences and variable relative humidity prevailing during ripening in different agroenvironments (e.g., coastal vs. inland plains and highlands), it also reflects the absence of detailed physicochemical and physiological characterizations of carob fruit ripening phenology. The protracted manual harvesting of nonsystematic orchards, the possibility of allowing for postharvest pod dehydration and the damage inflicted potentially on overlapping early blooms by fruit knocking tend to encourage an earlier harvest.

The turning of the pedicel from green to dark has been referred to as a practical sign of ripeness and readiness for harvesting carob fruit [3]. However, the establishment of harvest maturity indices lacks a well-defined reference base linked to the phenology of the ripening carob fruit. Attempts to define the physicochemical and physiological events underlying carob fruit ripening events have been scarce and fragmentary, referring mostly to compositional differences between the unripe and ripe fruits. Unripe carob fruit was found higher in phenolic content (phenolic acids, polyphenols, flavonoids and tannins) than the ripe fruit, as well as a higher in vitro antioxidant capacity [14,15,16]. Reducing sugars were in higher concentration in the unripe fruit, whereas sucrose was higher in the ripe fruit [17,18].

Changes in the carob’s metabolome during ripening may be interacted with by the effects of the genotype and the environment [14,15]. In order to define readily applicable harvest maturity indices, such changes must reference a consistent set of discrete ripening stages, identifiable visually (e.g., pod and seed coloration) and supported by fundamental physiological (e.g., respiratory activity) and physical (e.g., moisture content) determinations. Accordingly, the aims of the present study were to define critical stages for carob harvest maturity, ranging from fully developed fleshy green fruit to fully ripe, dark and dehydrated fruit, and to unravel the corresponding changes in the fruit’s nonvolatile metabolome and correlations between the bioactive molecules and in vitro antioxidant potential. Moreover, metabolic profiles were examined for two distinct carob genotypes and two environments of low and high altitudes in order to assess how these factors interact in the ripening process. Compositional determinations of high standards were performed, including multiple traits of nutritive, sensory and bioactive–antioxidant significance, such as minerals, organic acids, soluble sugars, phenolic compounds, the protein content and in vitro assays of radical scavenging and antioxidant capacities. The current study comprises a significant contribution to our understanding of the metabolic events underlying carob fruit ripening; it establishes a set of harvest maturity indices for carob and provides insight into the nutritive, sensory and bioactive–antioxidant implications of harvesting carobs at different stages along the ripening continuum of this underutilized crop.

## 2. Materials and Methods

### 2.1. Plant Material

**Figure 1:**
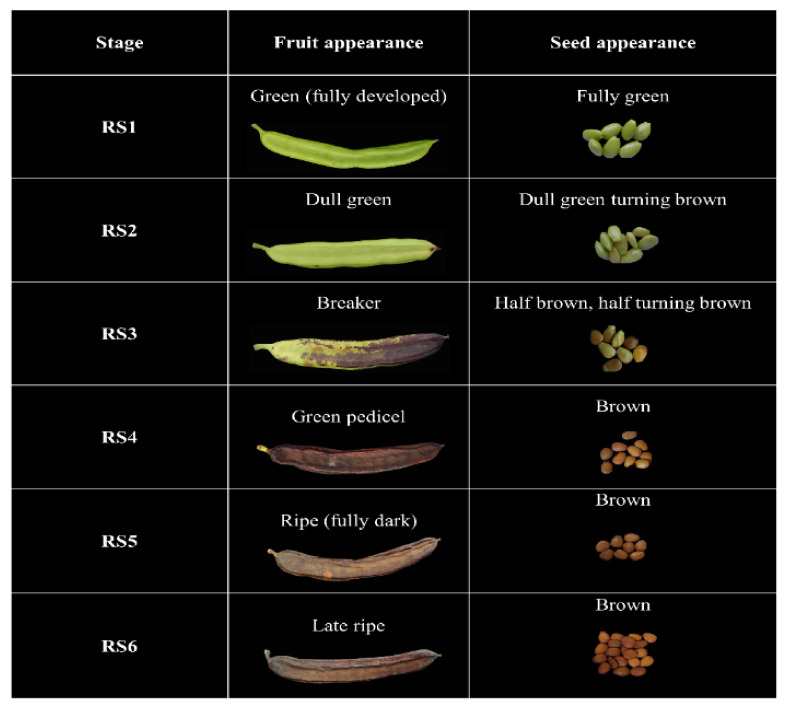
Description and illustration of fruit and seed colors corresponding to six critical carob fruit maturity stages.

Carob fruits were harvested from two genetically proximate phenotypic variants of the Cypriot carob (*Ceratonia siliqua* L.) landrace: Lefkaritiki (LF) and Mavroteratsia (MV) [19]. Fruits were sourced from individual, genetically identified and georeferenced trees at 15-m altitude (low altitude) in the coastal zone (Kalavasos) and from trees at 510-m altitude (high altitude) located in the inland mountainous zone (Vavla). These two localities were selected as representative of the two major agroenvironmental zones previously defined and modulating the significant effects on the compositional properties of the carob pulp [19]. The climatic conditions prevailing in these two localities are shown in Supplementary Table S1. Three replicate samples consisting each of six fruits were collected from the identified, georeferenced trees at each of the six critical harvest maturity stages (RS1–RS6). Description and visual representation of the six critical harvest maturity stages is presented in Figure 1. Harvest was performed in the morning hours (08:00–10:00), and the samples were directly transferred to the Postharvest Physiology Laboratory of the Agricultural Research Institute, where physical and respiratory measurements were performed within the day. Intact carob fruits were frozen at −40 °C before lyophilized at 0 °C for 48 h in a Christ, Alpha 1-4 lyophilizer (Osterode, Germany). The pods were coarsely ground in a Vita Prep 3 blender (Vita-Mix Corp., Cleveland, OH, USA) operated at low speed and deseeded. Coarse kibbles and seeds were then additionally lyophilized for about 24 h to constant weight. Fully desiccated kibbles were ground in a CT293 Cyclotech mill (Foss Analytical A/S, Hillerød, Denmark) to pass through a 1-mm sieve, and the powder obtained was stored at −60 °C for compositional analyses.

### 2.2. Fruit Respiration Rate, Moisture Content and Colorimetry of Pulp and Seed

Respiration rates were measured in a static system [20]. Samples of five intact, fresh fruits were weighed and sealed in 1.5-L glass jars equipped with ball valve-controlled outflows bearing 11-mm Thermogreen TM rubber septa (Supelco, Bellefonte, PA, USA). Jars were kept at 25 ± 0.5 °C in a Sanyo MIR153 (Panasonic, Osaka, Japan) incubator for 60 min (stages RS1–RS4) or 300 min (RS5 and RS6), and headspace samples were then taken with a hypodermic syringe. Respiration rate was measured on a portable gas analyzer equipped with an infrared sensor (CheckMateII; PBI Dansensor A/S, Rønnedevej, Denmark) and expressed in ml CO2 kg^−1^ h^−1^. Fruit moisture content was determined based on the fresh weight of the fruit, and the dry weight was obtained following lyophilization to a constant weight; both weights were measured on a Precisa XT120A analytical balance (Precisa Gravimetrics, Dietikon, Switzerland). The color component L* (lightness) of the carob powdered pulp was determined using a Minolta CR-410 Chroma Meter (Minolta, Osaka, Japan) and that of the seed using a Minolta CR-400, both equipped with a diffusion illumination 0° viewing angle geometry and the color space XYZ, Yxy, L*a*b*, Hunter, L*C*h, Munsell as the default.

### 2.3. Pulp Mineral and Protein Content

Pulp concentrations of cations (K+, Ca^2+^, Mg^2+^ and Na^+^) and anions (NO_3_^−^, PO_4_^3−^and SO_4_^2−^) were analyzed as previously described in detail by Rouphael et al. (2017) [21]. The cations were separated on an ion chromatograph (ICS-3000, Dionex, Sunnyvale, CA, USA) using a Dionex IonPac CS12A (4 × 250 mm) analytical column connected to a Dionex IonPac CG12A (4 × 50 mm) guard column. Anions were separated using a Dionex IonPac AS11-HC analytical column (4 × 250 mm) connected to a Dionex IonPac AG11-HC (4 × 50 mm) guard column. The concentrations of all minerals were expressed in g kg^−1^ dry weight (dw). The total protein content was assessed based on the nitrogen content according to the Kjeldahl method, using a nitrogen-to-protein conversion factor of 6.25 [22].

### 2.4. Soluble Carbohydrates and Organic Acids

The analysis of water-soluble carbohydrates was performed on aqueous extracts of the lyophilized fruit pulp clarified using the Carrez Clarification Kit (Sigma-Aldrich, St. Louis, MO, USA), as previously described in [23]. Soluble sugars (glucose, fructose and sucrose) were analyzed by liquid chromatography on an Agilent 1260 Series HPLC system (Agilent Technologies, Santa Clara, CA, USA) equipped with a Refractive Index detector and operated by Chem-Station software. Separation was performed through a Waters 4.6 × 250 mm carbohydrate column (Waters, Milford, MA, USA) at 35 °C using an acetonitrile:water (82:18) mobile phase at an isocratic flow rate of 1.5 mL min^−1^. Injection volume was 20 μL. Quantification was performed against calibration curves of fructose, glucose and sucrose external standards (0.2–2.0 g 100^−1^ mL) with a coefficients of determination R^2^ > 0.999. Results were expressed as mg g^−1^ dw.

Analysis of organic acids on lyophilized fruit pulp was performed as described in detail by Rouphael et al. (2017). Organic acids (malic, citric and oxalic) were analyzed on an ion chromatograph (ICS-3000, Dionex, Sunnyvale, CA, USA) equipped with an electrical conductivity detector. Separation of organic acids was facilitated by an IonPac AS11-HC analytical column (4 × 250 mm) fitted with an IonPac AG11-HC guard (4 × 50 mm) column. The concentrations of organic acids were expressed in mg g^−1^ dw.

### 2.5. Total Phenols and Condensed Tannins

The total phenols content (TPC) of the carob fruit pulp was extracted in methanol:H_2_O:acetic acid (80:19.5:0.5) in the dark at 4 °C for 24 h and determined as previously described by Kyriacou et al. (2016) based on a slight modification of the method of Singleton et al. (1999) [24]. Quantification was performed on a Jasco V-550 spectrometer (Jasco Corp., Tokyo, Japan), based on the absorbance at 765 nm, against a linear calibration (R^2^ > 0.99) established with gallic acid (50−500 mg L^−1^) as the external standard. Results were expressed as gallic acid equivalents (GAE) in g kg^−1^ pulp.

Condensed tannins (proanthocyanidins) were determined according to the vanillin methods of Sun [25] et al. (1998) and Sepperer et al. (2019) [26], modified as previously described in detail by Antoniou et al. (2020). Extraction of 0.5-g lyophilized pulp was performed in 10-mL methanol:H_2_O:HCl (50:40:10) for 24 h at 4 °C in the dark. Quantification in catechin equivalents (CE) was performed at 500 nm against calibration with catechin external standards (0.025–0.5% *w*/*v* in methanol) replacing the sample and methanol replacing the sample as blank. Results were expressed in mg CE g^−1^ dw.

### 2.6. Analysis of Polyphenols by UHPLC-Q-Orbitrap HRMS

Samples (0.3 g) of freeze-dried carob pulp were extracted with a 10-mL mixture of ethanol:water (80:20, *v*/*v*) using a slight modification of the procedure reported by Rached et al., 2016 [27]. Following intensive vortexing for 1 min and 30 min of sonication in the dark at room temperature, the extracts were centrifuged at 3000 rpm for 10 min, and the supernatant was filtered through 0.22-µm nylon filters (Phenomenex, Castel Maggiore, Italy), prior to injection into the UHPLC Orbitrap MS. Analysis was performed on an Ultimate 3000 UHPLC system (Thermo Fisher Scientific, Waltham, MA, USA) equipped with a Kinetex 1.7-μm biphenyl (100 × 2.1 mm) column (Phenomenex, Torrance, CA, USA) maintained at 25 °C. Injection volume was 2 μL, and flow rate was 0.2 mL min^−1^. A gradient elution program was applied based on water (A) and methanol (B), both containing 0.1% formic acid: 0 min—5% B, 1.3 min—30% B, 9.3 min—100% B, 11.3 min—100% B, 13.3 min—5% B and 20 min—5% B. The UHPLC system was coupled to a Q Exactive Orbitrap liquid chromatography tandem-mass spectrometry (LC–MS/MS). A heated electrospray ionization source (HESI II, Thermo Fischer Scientific) operating in negative ion mode (ESI-) was used. Ion source parameters were: sheath gas-flow rate 45 units, auxiliary gas-flow rate 10 units, spray voltage −3.5 kV, capillary temperature 275 °C, S-lens (RF) level 50 and auxiliary gas heater temperature 350 °C. Analysis scan events, MS data acquisition parameters and AIF scan conditions were previously described in detail [28]. Xcalibur software v. 3.1.66.10 (Xcalibur, Thermo Fisher Scientific) was used to perform data analysis and processing. Individual compounds were quantified using the corresponding external standard calibration curves. In the absence of corresponding standards, quantitation was carried out using standards of the same chemical group, demonstrating a similar response to the mass spectrometer.

### 2.7. In Vitro Antioxidant Activity Assays

The Ascorbate Equivalent Antioxidant Activity (AEAC) of lyophilized carob fruit pulp was assayed in vitro on methanolic extracts using the 1,1-diphenyl-2-picrylhydrazyl free-radical (DPPH) scavenging capacity assay [29]. Quantification was performed at 517 nm against ascorbate external standards (100–1000 μM) and expressed in ascorbate equivalents (AAE) as μmole ΑΑΕ g^−1^ dw. The in vitro antioxidant activity of the methanolic extracts was also assayed according to the ferric-reducing antioxidant power (FRAP) assay of Benzie and Strain (1996) [30]. Quantification was performed at 593 nm against external standard calibration curves of ascorbic acid at 100–1000μΜ. Results were expressed in ascorbate equivalents as μmole ΑΑΕ g^−1^ dw.

### 2.8. Statistical Analysis

Statistical analysis was performed using the SPSS statistical package (IBM, SPSS ver. 25; Armonk, NY, USA). Data were subjected to three-way analysis of variance (ANOVA), and the results are presented as the percentage of variance of the Sum of Squares (SS) explained by the main effects and their interactions. Mean comparisons between ripening stages were performed by Tukey’s b test. Means of the main effects are presented with their standard errors. Pearson correlations on treatment mean values were calculated to explore relations between traits. Regression plots on treatment mean values are presented for selected traits. Traits were regressed on one or two factors depending on the percentage of variance explained by the main effects.

## 3. Results and Discussion

### 3.1. Critical Stages for Carob Harvest Maturity

In order to map the changes in carob fruit metabolome during ripening, a set of universal harvest maturity indices or stages must be defined that comply with two major requirements: (a) they should be discernible through physical characteristics readily perceptible and applicable in situ, and (b) they must correspond to critical morphophysiological events in the ontogeny of fruit ripening. In the present work, six critical harvest maturity stages were defined that corresponded to principal changes in fruit and seed coloration. These stages ranged from RS1 (fully developed green fruit bearing green seeds) to RS6 (fully dark late ripe fruit bearing brown seeds). Full descriptions of the fruit and seed appearances and visual representations of the six harvest maturity stages are presented in Figure 1.

**Figure 2:**
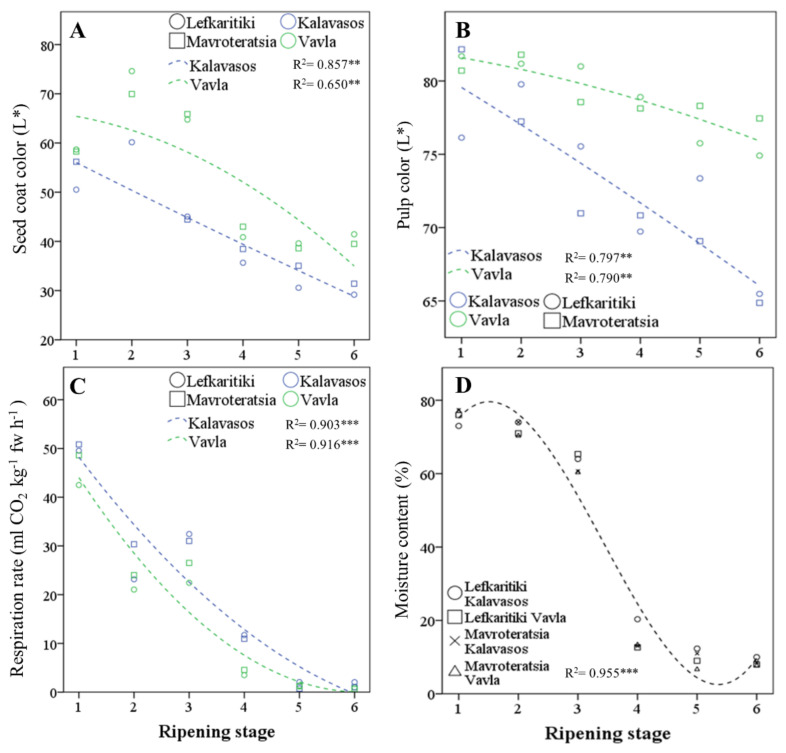
Regressed change in (**A**) seed coat color parameter L*, (**B**) pulp color parameter L*, (**C**) respiration rate and (**D**) moisture content of carob fruits at six critical stages of maturity, from fully developed green (RS1) to late ripe (RS6) fruit. fw: fresh weight. Significant effect at the *p* ≤ 0.01 (**) and *p* ≤ 0.001 (***) levels

Conventional harvesting of carobs throughout the Mediterranean commences at RS5, when the entire surface of the fruit turns dark, and the abscission zone is fully developed [3]. As opposed to commercial maturity, however, physiological maturity is signaled by the complete coloration of the seeds to brown and the attainment of germinability, while the pedicel still remains green (RS4). The ripening stage and location significantly affected the CIELAB parameter L* (lightness) of the seed coat, whereas the effect of the genotype was nonsignificant. ([31] Table 1 and Figure 2A). The seed coat in both genotypes darkened with progressive maturity before stabilizing at RS5. The seeds of fruits obtained from a higher altitude (Vavla) retained a lighter color than the seed from a lower altitude (Kalavasos) throughout ripening. The transition in pulp color L* was slower than that observed for seed color (Table 1 and Figure 2B). The pulp color was lightest (highest L*) at RS1 and RS2, intermediate at RS3–RS5 and darkest at RS6. It is noteworthy that the pulp color darkened significantly from RS5 to RS6, although both the external color of the fruit and seed color assumed their ultimate dark color at RS5. The lightness of the pulp color is a practical index of carob quality widely acknowledged by kibbling plants but not yet given adequate attention by researchers as a potentially quantifiable index of carob quality (personal communication). The current work demonstrated that the pulp color generally is mainly defined by the ripening stage and influenced by altitude, without a significant effect exerted by the genotype. Whereas commercial maturity is signaled by the turning of the pedicel from green to dark [3], for practical reasons, the harvest period extends until September, when the fruit reaches late maturity (RS6). However, the pulp color continues to darken after the fruit assumes a completely dark and desiccated appearance (RS5), despite the fact that the fruit moisture content and respiration rate reach a steady low at RS5. It is likely that the carob pulp darkening in late maturity results from the enzymatic oxidation of polyphenols by polyphenol oxidase (PPO) and peroxidase (POX). The activity of these ubiquitous enzymes reduces the quality of horticultural products and results in the loss of color through the production of orthoquinones that form dark-colored melanin pigments, which polymerize nonenzymatically. The oxidation of polyphenols is exacerbated by the stressful conditions of heat, light and moisture that may also reduce the overall nutraceutical value of the carob fruit [32]. This is further supported by the absence of a significant genotype effect on the pulp color, as opposed to location, with fruit from the higher altitude retaining a higher pulp L* throughout ripening. It is likely that the higher relative humidity prevailing in coastal regions, such as Kalavasos (Supplementary Table S1), is linked to the darker fruit pulp color attained. It may be thus concluded that fruit from inland mid-altitudinal areas harvested at the onset of full coloration (RS5) are more likely to carry a desirably light-colored pulp.

Major changes were observed throughout ripening with respect to the rate of fruit respiration and the fruit moisture content, which constitute the key physiological parameters of maturity (Figure 2C). The turning point for both these parameters was the transition from RS3 (half-colored fruit surface and seeds) to RS4 (green pedicel and brown seeds), during which the mean respiration rate declined from 28.1 to 7.7 mL CO_2_ h^−1^ kg^−1^ at 25 °C (72.6% decline), and the moisture content declined from 62.6% to 14.8% (76.4% decline; Figure 2D and Table 1). A smaller but, however, significant decline in both parameters was noted up to RS5, wherein the fruit colored fully and assumed the desiccated texture suitable for long-term storage. The fruit moisture content stabilized at a low of 8.78–9.75% at commercial maturity (RS5 and RS6), which is similar to the levels reported by Vekiari et al. (9.01% ± 0.99%; 2012) [17] and Othmen et al. (9.03% ± 11.30%; 2019) [18] but higher than those of Musa Özcan et al. (6.01% ± 0.11%; 2007) [33]. Moisture contents in carob pulp as high as 19.18% have been reported [34], which are likely due to premature harvest. At RS5 and RS6, the respiration rate dropped below 5 mL CO_2_ kg^−1^ h^−1^, which ranks ripe carob fruit as a very low respiring horticultural commodity, along with dates, nuts and dehydrated fruits and vegetables, predisposed for long-term storage, with a low production of vital heat [35,36]. Variations in the respiration rate and fruit moisture content were almost entirely controlled by the ripening stage, which determined 96.3% and 99.1% of the total variance for the two parameters, respectively. Location and genotype determined cumulatively less than 2% of the total variance for the same parameters, which highlights the universality of the critical harvest maturity stages defined herein. Greater variation in the moisture content could possibly be observed between localities differing in precipitation levels, as reported by Othmen et al. (2019) [18] for semi-arid (13.73–14.27%) and arid localities (9.03–11.30%) in Tunisia, with a possible uptake of moisture directly from the air in humid environments.

### 3.2. Mineral and Protein Content

Fruits and vegetables facilitate the dietary intake of K, Mg, Na, P and Ca by roughly 35%, 24%, 11%, 11% and 7%, respectively [37]. The uptake of macro-minerals through the human diet is critical for preventing nutritional disorders and chronic disease or fatigue owing to their well-established functionalities in the body homeostasis and metabolism [38]. In the current work, the maturity stage affected significantly the concentrations of all macro-minerals in the carob pulp, which were found in the following decreasing order throughout ripening: K > Ca > Mg > P (Table 2). All macro-minerals declined in concentrations with the progressive maturity, except for Ca, which climaxed between RS2 and RS3 and declined thereafter. An analogous decline for K and Mg was found by Othmen et al. (2019) for Tunisian carobs (unspecified cultivar) during ripening, roughly defined as unripe, mid-ripe and ripe. The location had a significant effect on the Ca and P concentrations, with Ca being higher in Vavla and P in Kalavasos. The genotype had a major contribution to the variance of Mg and, especially, K concentrations, with “Lefkaritiki” having higher K and “Mavroteratsia”, higher Mg. The present data corroborate the importance of the carob fruit as a concentrated source of potassium. In its late ripe stage (RS6), the carob pulp had a mean potassium content of 11.22 ± 0.63 mg g^−1^ dw, which approximates the content of banana fruits (14.27 mg g^−1^ dw), widely acknowledged as a rich dietary source of potassium [39]). The strong genotype effect on K concentration is reflected in the slightly lower concentrations reported by Kokkinofta et al. (2020) for carob genotypes sourced from Cyprus and Italy (9.03 and 10.33 mg g^−1^ dw) and the two-fold higher content (24.7 mg g^−1^ dw) found by Musa Özcan et al. (2007) in Turkish carobs. Similarly, the genotype effect on Mg concentrations is evident not only among the genotypes currently reported, which are comparable to those found in Jordanian carobs (0.82 mg g^−1^ dw [34]) but, also, in comparison to Turkish genotypes that present significantly higher Mg content [33]. Calcium was the second-most abundant macro-mineral found in carob pulp in concentrations 2.21 ± 0.21 mg g^−1^ dw, similar to those (2.38 mg g^−1^ dw) reported by Kokkinofta et al. (2020) but half of those reported for Turkish carobs (4.21 mg g^−1^ dw [33]). The present findings contributed towards establishing a reference base for the nutritional value of carob fruits in terms of the mineral content, information on which remains scarce.

The protein content of the pulp was determined chiefly by the ripening stage and location, with the genotype controlling just 3.2% of the variance (Table 2). The protein content declined with the progressive ripening, following an analogous pattern that was previously reported for Greek and Turkish carobs [17,40]. The most substantial reduction (30.8%) was observed between RS1 and RS3, signaled by the onset of external fruit coloration. The protein content stabilized after the onset of physiological maturity (RS4) and ranged as 4.14–4.59% dw thereafter. A similar protein content was reported by Avallone et al. (1997) for Sicilian carobs, by Kokkinofta et al. (2020) for Cypriot carobs among a wider ranging (2.29–5.91% dw) of genotypes of various Mediterranean origins, by Musa Özcan et al. (2007) for Turkish carobs (4.71% ± 0.66%) and by Vekiari et al. (2012) for Greek wild genotypes (4.10% ± 0.15%) but not for select genotypes (6.4% ± 0.18%). However, the present analysis of variance for the protein content corroborates that the protein content may vary more by location than genotype, wild genotypes excluded. Altitude seems an important environmental parameter in this respect, as throughout ripening, the protein content was maintained higher (5.96% ± 0.19% dw) in the fruit harvested at a lower altitude (Kalavasos) compared to fruit from a higher altitude (Vavla; 4.30% ± 0.21% dw).

### 3.3. Soluble Carbohydrates and Organic Acids

The carob pulp has been chiefly exploited for its rich sugar content. In the current work, variations in the total sugars of the pulp were determined almost entirely by the ripening stage, which accounted for 91.7% of the variance, while the effects of the location and genotype, as well as interactions, were nonsignificant (Table 3). The total sugars increased with the progressive ripening, marking a two-fold increase from RS1 to RS6 to reach a maximal mean content of 498.7 mg g^−1^ dw. The most critical stages for sugar accumulation were the onset of fruit coloration (RS2 to RS3) and the transition from the breaker to the green pedicel stage (RS3 to RS4). The relative increases in total sugars at these transitional stages amounted to 42.5% and 33.8%, respectively. The subsequent relative increase was much slower; however, it is worth noting that the sugar accumulation, expressed on a dry weight basis, culminated at the late ripe stage (RS6). It is likely that the ripening carob fruit continues to function as a sink and draws sugars from the photosynthetic sources of the tree at least up to the green pedicel stage (RS4) and, possibly, up to the later stages, with the degradation of hydrolyzable tannins in the fruit adding also to the carbohydrate balance at stages RS5 and RS6 (Table 4; see Section 3.4. below for further information).

The mean total sugars content attained at commercial maturity by the two select genotypes examined in the present study (Table 3) was similar to that reported for Turkish carobs (48.35% dw [33]), higher than those reported for Greek carobs (43.3% dw [17]) and lower only to those found by Kokkinofta et al. (2020) as a mean of various Cypriot genotypes (54.15% dw). The sugar contents among eight Sicilian genotypes ranged widely (38–50% dw [5]). The above variation in the total sugar content is in-line with the range (40–56% *w*/*w*) reported by Batlle and Tous (1997). Variation may stem from agroenvironmental and genotypic differences, particularly between wild and select genotypes and, possibly, from the alternate bearing pattern common to carob trees that usually results in a yield and sugar depression in the “off year” [17,19,41]. It is worth noting that total sugars in the present study increased up to the late ripe stage (RS6—end of August), whereas, in overripe Turkish carobs collected at the end of October, the sugar content decreased with respect to earlier harvests [40]. However, a lack of genotype identification and established maturity indices humpers the drawing of reliable conclusions from the above studies as opposed to the present.

The variations in the concentrations of sucrose and glucose with respect to the ripening stage were significant, whereas the variation in fructose was nonsignificant (Table 3). As opposed to the ripening stage, the location and genotype had nonsignificant or practically negligible effects. Glucose decreased with ripening and incurred its sharpest drop from RS3 to RS4; after which, it stabilized at about 60% of its original concentration. Conversely, sucrose marked a near six-fold increase from RS1 to RS6, with the sharpest increase also noted during transition from the breaker (RS3) to the green pedicel stage (RS4). The ratio of reducing sugars to sucrose is indicative of the transformation in sugar composition during ripening, with a maximal ratio observed at RS1 (3.45 ± 0.32) and a minimal ratio attained at RS6 (0.41 ± 0.02). An analogous transformation of this ratio from 3.45 to 0.41 corresponding to immature and mature carob pods was reported by Vekiari et al. (2012). It is apparent from the current results that the variation in sugar content among selected carob genotypes clonally propagated through grafting at different locales is limited, whereas the maturity at harvest is the critical factor.

The acid composition and concentration in carob pulp has received scant attention from previous workers, as sugars are the dominant sensory driver of carob fruits. However, the presence of organic acids even at minimal levels can influence the sensory profile of horticultural products, such as the sensation of freshness [41]. The effect of harvest maturity on the concentrations of the dominant acids (malate > citrate > oxalate) and total organic acids in the carob fruit pulp outweighed those of the location and genotype (Table 3). The predominance of malic acid and the decline of organic acids in ripe carob fruits was also reported in Egyptian carob genotypes [15]. Unlike the relative ratios of soluble carbohydrates, which varied with ripening, the ratio of major acids remained relatively stable at about 20:4:1 as malate:citrate:oxalate, despite the 50% reduction in total acids incurred from RS1 to RS6. Higher total acid concentrations (13.0 ± 0.6 mg g^−1^) were found in carob fruits harvested at a higher elevation (Vavla) as opposed to a lower elevation (10.9 ± 0.7 mg g^−1^; Kalavasos). Additionally, the total acids were higher in Lefkaritiki (13.0 ± 0.7 mg g^−1^) than in Mavroteratsia (10.9 ± 0.6 mg g^−1^). It is worth noting that the concurrent 50% increase in total sugars and 50% decrease in total acid concentrations during the transition from RS1 to RS6 translated into a four-fold increase in the sugar/acid ratio of the fruit, which signaled a significant change in the organoleptic properties [41]. Moreover, although the location had a nonsignificant effect on the sugar content, the higher acid content of fruit from a higher altitude amplified the differences in the sugar/acid ratios, resulting in a higher ratio at the lower elevation.

### 3.4. Analysis of Polyphenols by UHPLC-Q-Orbitrap HRMS

The pulp of commercially mature carob fruit is reportedly rich in phenolic acids, gallotannins and flavonoids [2]. However, changes in the carob fruit secondary metabolome during ripening have so far received scant attention. The consideration of bioactive constituents in determining the optimal harvest maturity for carob fruit has been largely overlooked, as harvesting carobs at their lowest moisture content is the chief interest of kibbling plants. The integration of MS and MS/MS spectra investigated by UHPLC-HRMS Orbitrap presently enabled the identification of 18 different phenolic compounds across the carob ripening stages (Table 4). All of the studied analytes exhibited better fragmentation patterns, producing the quasi-molecular ion (M−H)−. After a full-scan analysis, the accurate mass of the characteristic ions (precursor ions) was included in an inclusion list. Full-scan HRMS data acquisition captured all sample data, enabling the identification of untargeted compounds and retrospective data analysis without the need to rerun samples. The confirmation of the structural characterization of the untargeted analytes was based on the accurate mass measurement, elemental composition assignment and MS/MS spectrum interpretation.

**Figure 3:**
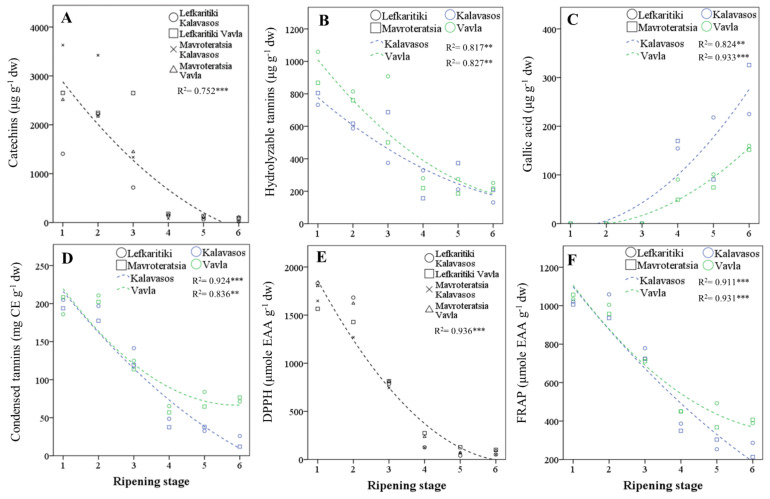
Regressed changes in the carob fruit pulp contents in (**A**) catechins, (**B**) hydrolyzable tannins, (**C**) gallic acid, (**D**) condensed tannins, (**E**) 1,1-diphenyl-2-picrylhydrazyl (DPPH) free-radical scavenging capacity and (**F**) FRAP (ferric-reducing antioxidant power) of carob fruits at the six critical stages of maturity from fully developed green (RS1) to late ripe (RS6) fruits. CE: catechin equivalents and dw: dry weight. Significant effect at the *p* ≤ 0.01 (**) and *p* ≤ 0.001 (***) levels.

The most abundant polyphenols by category were gallocatechin (catechins), naringenin-diglucoside (flavone glycosides), kaempferol-3-*O*-glucoside (flavonol glycosides), tetragalloyl-glucose (hydrolyzable tannins) and gallic acid (phenolic acids) (Table 5). The relative abundance of the main phenolic categories varied widely with the maturity stage, as opposed to location and genotype. Catechins and hydrolyzable tannins were the dominant phenolic categories present at the early stages of ripening (RS1–RS3), up to the onset of fruit coloration. The transition from the breaker stage (RS3) to the green pedicel stage (RS4) was marked by a stark decline (90.9%) in catechins, as well as a significant drop in hydrolyzable tannins (60.2%) and flavonol glycosides (52.1%), as opposed to the flavone glycosides that remained relatively stable (Figure 3A–C). Concomitant to the sharp drop in catechins, hydrolyzable tannins and flavonol glycosides during transition from RS3 to RS4 was the rise in gallic acid (3,4,5-trihydroxybenzoic acid). The predominance of gallate among phenolic acids in mature carob kibbles was also identified by previous workers [15,42]. Remarkably, gallic acid was near absent from stages RS1–RS3; it emerged at RS4 and nearly doubled in concentration by the late ripe stage (RS6). These changes unravel significant episodes in the ripening-related secondary metabolism of the carob fruit. The cleavage by hydrolytic enzymes of tetra-, tri-, and digalloyl glucose tannins abundant in immature fruits putatively fueled the rise observed in gallic acid by releasing the glucose and gallic acid units [43,44,45]. The loss of hydrolyzable tannins during fruit ripening is associated with a reduced astringency and undesirable mouth-feel sensation [41]. Moreover, the dissipation of glucose moieties from hydrolyzed gallotannins may support the carbohydrate balance of the developing fruit and indirectly contribute to sucrose synthesis in the later stages of ripening (RS4–RS6; Table 3). Such a precipitous decline in hydrolyzable tannins with progressive ripening has been observed in other tannin-rich fruits [43,46,47]. It has been further hypothesized that hydrolyzable tannins may polymerize during ripening and bind to proteins, cell wall polysaccharides and fibers, which limits their extractability and astringency [47]. Further research is thus warranted on the transformation of hydrolyzable tannins in developing carob fruits, notwithstanding enzymatic studies that are complicated by the protein precipitant properties of the substrates [48].

Catechins, hydrolyzable tannins and flavone glycosides were, overall, more abundant in fruits harvested at the higher elevation, whereas flavonol glycosides in total were not significantly affected by location (Table 5). In contrast to catechins, hydrolyzable tannins and flavone glycosides, the concentration of gallic acid from RS3 through RS6 was steadily higher in the fruits collected from the lower elevation, being, on average, near double in concentration compared to the higher elevation. Previous studies on strawberries have also highlighted the potentially significant effect of different environments on the tannin composition, particularly on the hydrolyzable/condensed tannins ratio [49]. Finally, the overall contribution of genotype to the variance of polyphenols was limited, with the exception of flavonol glycosides (Table 5). The differences between the two genotypes were most pronounced for flavonol glycosides, which had a near two-fold higher concentration in Mavroteratsia than Lefkaritiki. The total concentration of catechins was also higher by 20.6% in Mavroterstasia than Lefkaritiki. Conversely, the latter genotype had moderately higher concentrations of hydrolyzable tannins and gallic acid. Analogous genotype-dependent variations in the compositional profiles of hydrolyzable tannins were previously found in the tannin-rich fruits of *Phyllanthus emblica* [46].

### 3.5. Total Phenolics and Condensed Tannins

The content of the carob pulp in total phenolics and condensed tannins impacts the bioactive properties and organoleptic profile of the carob fruit pulp [2]. Their antioxidant capacity is related to the presence of hydroxyl groups and to the double-bond conjugation and resonance effects [50]. In the current study, both the total phenolics and condensed tannins declined substantially over ripening, but the decline was more pronounced for phenolics (90.6%) than tannins (76.6%; Table 6 and Figure 3D). An analogous decline in TPC (91.6%) from the immature to fully mature stage was reported for Greek carobs [17] and for Algerian carobs [14,16]. The sharpest fall in TPC and condensed tannins was observed after the onset of fruit coloration, particularly during the transition from the breaker (RS3) to green pedicel (RS4) stage. The TPC reached a minimum concentration at RS5 (17.4 ± 2.0 mg GAE g^−1^ dw) and remained unaltered through RS6. Thus, the pulp of Cypriot carob genotypes at full commercial maturity had comparable TPC to the mean content previously reported for cultivars from Italy (16.0 ± 2.1 mg GAE g^−1^ dw) [5], Portugal (19.0 ± 3.0 mg GAE g^−1^ dw) [51] and Greece (18.0 ± 0.8 mg GAE g^−1^ dw) [17]. The TPC reported in different studies, however, can vary significantly, and variations can be traced to differences in the genetic material, ripeness and agroenvironmental conditions [2]. Moreover, solvents of increasing polarity are likely to increase the yield of extractable TPC [16,52,53].

Unlike the TPC, the condensed tannins content was reduced by 15.2% from the ripe (RS5) to the late ripe (RS6) stage and, further, to the sharp drop (58%) observed at RS4. Thus, a significant reduction in condensed tannins can occur even after the carob fruit attains complete coloration. The levels of condensed tannins (46.45 ± 8.50 mg CE g^−1^ dw) present in the Cypriot carob genotypes at the late ripe stage (RS6) were much higher than those previously reported for Italian [5] and Portuguese carobs [51]. However, the variations among the carob genotypes in condensed tannins can be very high, as, for instance, between Portuguese cultivars Preta de Lagos (1.1 ± 0.1 mg CE g^−1^) and Costela (10.0 ± 0.3 mg CE g^−1^ [51]. Significant variations may also derive from different sampling environments, extraction solvents of differing acidity and quantification methods showing distinct specificities [54]. The levels of condensed tannins presently reported in the acidified methanolic extracts of mature carobs enhance the bioactive value of this fruit and can be considered comparable to those found in cider [54]. Both the total phenolics and condensed tannins were found in higher concentrations in the fruits harvested from the higher elevation. This difference was more pronounced for tannins (19.2%) than phenolics (7.5%), which highlights the variability of these constituents elicited by different agroenvironments. Both classes of compounds were also found at higher levels in Lefkaritiki than Mavroteratsia. The effects of location and genotype on both variables were nonetheless marginal compared to that of harvest maturity.

### 3.6. In Vitro Antioxidant Activity and Correlation with Polyphenols

Variances in the DPPH and FRAP results were controlled almost entirely (>95%) by the harvest maturity stage (Table 6). Both assays showed declining antioxidant capacity of the pulp extracts with ripening, with the sharpest drop occurring in the transition from RS3 to RS4 (Figure 3E,F). The minimum levels of antioxidant capacity were obtained at RS5 for FRAP and RS6 for DPPH, although the latter demonstrated a faster decline over ripening than the former. Similar changes in the DPPH scavenging capacity were found in unripe, mid-ripe and ripe Algerian carobs of wild and cultivated genotypes [14,16]. The genotype had no effect on DPPH, while the FRAP levels were higher in Lefkaritiki than Mavroteratsia. As previously observed for the total phenolics and tannins, both the FRAP and DPPH capacities were higher in the fruits obtained at the higher elevation. This variation reflects the plasticity of bioactive secondary metabolites in mediating abiotic stresses related to the edaphoclimatic parameters of different environments [55].

The total concentration of phenolic compounds found in carob fruit pulp was highly correlated with both the ferric-reducing antioxidant power (FRAP; r = 0.988; *p* < 0.01) and the free-radical scavenging capacity (DPPH; r = 0.982; *p* < 0.01; Table 5). A significant correlation of the radical scavenging capacity with the phenolic content was also reported for the Algerian variety Lahlou (r = 0.95, *p* < 0.05; [16]. The correlation between the two antioxidant assays was also high and significant (r = 0.977; *p* < 0.01). Among the major categories of polyphenols detected and quantitated, the highest correlation factors for the FRAP and DPPH antioxidant activities were obtained with condensed tannins (r = 0.988 and r = 0.969; *p* < 0.01), catechins (r = 0.886 and r = 0.876; *p* < 0.01) and hydrolyzable tannins (r = 0.881 and r = 0.876; *p* < 0.01). Significant but lower correlation factors (r = 0.695 and r = 0.688; *p* < 0.01) were also obtained for flavonol glycosides. Through their electron-donating properties, all polyphenols were able to scavenge reactive oxygen species and generate more stable phenoxyl radicals [54]. The hydrolyzable tannins, however, were more efficient scavengers than the catechins and demonstrated a strong radical scavenging capacity through galloylation, the oxidative coupling of neighboring galloyl groups [48]. Gallotannins have been attributed to in vivo anti-inflammatory [56], whereas catechins have been valued as protective, agents against diseases caused by oxidative stress [57]. The high presence of both prior to physiological maturity renders immature carob fruits a potent source of bioactive phytochemicals. Among the individual polyphenols, the highest correlation factor was obtained for epigallocatechin-gallate (r = 0.920 and r = 0.900; *p* < 0.01). The sharp drop in both hydrolyzable tannins and catechins demonstrated particularly at the breaker to green pedicel stages of carob ripening thus reduced correspondingly the antioxidant capacity of the mature carob fruits. Gallic acid also presents a significant radical scavenging potential and is mainly responsible for the antioxidant capacity of mature carob fruit pulp [58]. However, the precipitous drop in hydrolyzable and nonhydrolyzable tannins and catechins abundant in carob fruits before the critical green pedicel stage (RS4) compromised the in vitro antioxidant capacity but configured a palatable organoleptic profile thereafter.

## 4. Conclusions

The present study demonstrated that changes in the carob fruit primary and secondary metabolomes and its postharvest antioxidant potential are far wider between the successive stages of harvest maturity than between the genotypes and locations. The major metabolic events during carob ripening take place between the onset of fruit coloration (breaker) and the green pedicel stage, which signals physiological maturity. These included a precipitous decline in the fruit respiration rate, moisture content, total phenolic content, condensed tannins, hydrolyzable tannins, catechins and flavonol glycosides, which resulted in the loss of radical scavenging and antioxidant capacity from the fruit pulp extracts. The loss of hydrolyzable and nonhydrolyzable tannins and catechins, however, reduced the astringency and configured a palatable organoleptic fruit profile at full maturity. Physiologically immature carob fruits, however, merit attention as a potent source of gallocatechins, known for their in vivo action as anti-inflammatory and antihyperlipidemic agents, and of catechins, which may act as cytotoxic agents. In that respect, the harvest of carobs soon after the onset of physiological maturity constitutes an optimal combination of functional and sensory qualities.

## Figures and Tables

**Table 1:** Analysis of variance, percentage of total variance (PTV) and means ± standard error (SE) for the rates of respiration, moisture content, pulp and seed color lightness (L*) of carob fruits derived from two locations (altitudes) and two genotypes at six critical stages of maturity. RS1–RS6: the six critical harvest maturity stages.

Source of Variance	Respiration		Moisture		L*—Pulp		L*—Seed	
	PTV	Sig	PTV	Sig	PTV	Sig	PTV	Sig
Maturity (M)	96.30	***	99.07	***	43.30	***	71.15	***
Location (L)	1.39	***	0.08	**	35.50	***	13.29	***
Genotype (G)	0.18	**	0.05	ns	0.07	ns	0.09	ns
M × L	0.68	***	0.12	*	8.74	***	5.31	***
M × G	0.41	**	0.13	ns	3.30	***	0.37	ns
L × G	0.06	ns	0.00	ns	0.27	ns	0.28	*
M × L × G	0.21	*	0.09	*	4.65	***	0.32	ns
Error	0.77	ns	0.45	ns	4.17	ns	9.19	ns
Means								
Maturity	(ml CO_2_ kg^−1^ h^−1^)		(%)		(0–100)		(0–100)	
RS1	47.86 ± 1.27 a		75.83 ± 0.59 a		80.17 ± 0.84 a		55.91 ± 0.98 b	
RS2	24.64 ± 1.21 c		72.33 ± 0.57 b		79.99 ± 0.64 a		68.23 ± 1.38 a	
RS3	28.10 ± 1.25 b		62.58 ± 0.69 c		76.52 ± 1.15 b		55.02 ± 1.76 b	
RS4	7.68 ± 1.12 d		14.83 ± 1.60 d		74.40 ± 1.26 c		39.49 ± 0.57 c	
RS5	1.33 ± 0.16 e		9.75 ± 0.91 e		74.12 ± 1.06 c		35.95 ± 0.71 d	
RS6	1.17 ± 0.23 e		8.58 ± 0.29 e		70.68 ± 1.72 d		35.38 ± 0.98 d	
Location								
Kalavasos (15 m)	20.49 ± 3.00 a		41.53 ± 4.99 a		72.93 ± 0.90		41.51 ± 1.06 b	
Vavla (510 m)	16.44 ± 2.76 b		39.78 ± 5.17 b		79.03 ± 0.41		52.99 ± 1.33 a	
Genotype								
Lefkaritiki	17.73 ± 2.76 b		41.31 ± 5.01 a		76.12 ± 0.82		47.59 ± 1.41	
Mavroteratsia	19.20 ± 3.03 a		40.00 ± 5.15 b		75.84 ± 0.91		47.33 ± 1.26	

Data represent three replicates, each consisting of six fruits, for each treatment. ns = nonsignificant effect; significant effects at the *p* ≤ 0.05 (*), *p* ≤ 0.01 (**) and *p* ≤ 0.001 (***) levels. Different letters within each row per effect indicate significant differences according to Tukey’s b test (*p* < 0.05).

**Table 2:** Analysis of variance, percentage of total variance and means ± SE for potassium (K), magnesium (Mg), calcium (Ca) phosphorous (P) and protein concentration in the pulp of carob fruits derived from two locations (altitudes) and two genotypes at six critical stages of maturity. dw: dry weight.

Source of Variance	K		Mg		Ca		P		Protein	
	PTV	Sig	PTV	Sig	PTV	Sig	PTV	Sig	PTV	Sig
Maturity (M)	17.4	***	52.2	**	21.2	***	26.4	***	53.4	***
Location (L)	2.0	*	1.8	*	26.3	***	20.7	***	33.5	***
Genotype (G)	37.4	***	10.8	***	7.7	**	9.0	***	3.2	***
M × L	7.4	**	3.4	ns	11.3	**	7.2	***	2.8	**
M × G	5.9	**	6.0	*	7.4	*	5.6	**	1.5	*
L × G	5.5	**	0.3	ns	2.3	*	15.4	***	0.6	*
M × L × G	8.1	**	4.9	ns	3.0	*	5.4	**	0.9	ns
Error	16.3		20.6		20.8		10.3		4.3	
Means										
Maturity	(mg g^−1^ dw)		(mg g^−1^ dw)		(mg g^−1^ dw)		(mg g^−1^ dw)		(% dw)	
RS1	14.09 ± 072 ab		1.60 ± 0.06 a		2.32 ± 012 c		0.59 ± 0.04 a		7.11 ± 0.29 a	
RS2	14.46 ± 0.93 a		1.43 ± 0.04 b		2.95 ± 0.32 ab		0.50 ± 0.07 b		5.81 ± 0.39 b	
RS3	12.82 ± 066 bc		1.42 ± 0.08 b		3.47 ± 0.41 a		0.4 0 ± 0.03 c		4.92 ± 0.28 c	
RS4	13.62 ± 059 abc		1.21 ± 0.07 c		2.43 ± 022 bc		0.36 ± 0.03 c		4.14 ± 0.27 d	
RS5	12.58 ± 065 c		1.14 ± 0.04 cd		2.38 ± 0.16 bc		0.39 ± 0.04 c		4.59 ± 0.31 c	
RS6	11.22 ± 063 d		1.02 ± 0.05 d		2.21 ± 0.21 c		0.37 ± 0.04 c		4.21 ± 0.22 d	
Location										
Kalavasos (15 m)	13.50 ± 052 a		1.27 ± 0.05 b		2.13 ± 0.11 b		0.51 ± 0.03 a		5.96 ± 0.19 a	
Vavla (510 m)	12.77 ± 033 b		1.34 ± 0.04a		3.12 ± 0.17 a		0.36 ± 0.02 b		4.30 ± 0.21 b	
Genotype										
Lefkaritiki	14.71 ± 040 a		1.21 ± 0.04 b		2.36 ± 0.15 b		0.48 ± 0.03 a		4.87 ± 0.23 b	
Mavroteratsia	11.55 ± 028 b		1.39 ± 0.05 a		2.90 ± 0.16 a		0.38 ± 0.02 b		5.38 ± 0.25 a	

Data represent three replicates, each consisting of six fruits, for each treatment. ns = nonsignificant effect; significant effect at the *p* ≤ 0.05 (*), *p* ≤ 0.01 (**) and *p* ≤ 0.001 (***) levels. Different letters within each row per effect indicate significant differences according to Tukey’s b test (*p* < 0.05).

**Table 3:** Analysis of variance, percentage of total variance and means ± SE for glucose, fructose, sucrose, total sugars, malate, citrate, oxalate and total organic acid concentrations and the ratios of reducing sugars/sucrose and sugars/acids in the pulp of carob fruits derived from two locations (altitudes) and two genotypes at the six critical stages of maturity.

Source of Variance	Glucose		Fructose		Sucrose		Total Sugars		Malate		Citrate		Oxalate		Total Acids		Reducing/Sucrose		Sugars/Acids	
	PTV	Sig	PTV	Sig	PTV	Sig	PTV	Sig	PTV	Sig	PTV	Sig	PTV	Sig	PTV	Sig	PTV	Sig	PTV	Sig
Maturity (M)	76.2	***	8.0	ns	96.0	***	91.7	***	71.1	***	50.6	***	51.5	***	72.1	***	86.2	***	78.9	***
Location (L)	0.1	ns	3.9	ns	0.0	ns	0.1	ns	9.0	***	0.7	*	14.4	***	6.3	***	0.5	**	6.1	***
Genotype (G)	1.1	ns	2.9	ns	0.5	**	0.6	*	5.4	***	9.4	***	0.6	ns	6.3	***	1.0	***	2.9	***
M × L	1.1	ns	5.9	ns	0.2	ns	0.3	ns	1.5	ns	16.1	***	4.4	*	2.6	*	5.4	***	4.3	***
M × G	8.2	***	16.4	*	0.3	ns	0.8	ns	2.5	*	1.6	ns	6.1	**	2.3	*	2.6	***	1.0	ns
L × G	1.0	ns	2.6	ns	0.0	ns	0.0	ns	0.0	ns	10.0	***	3.1	**	0.2	ns	0.1	ns	0.3	ns
M × L × G	0.9	ns	3.7	ns	0.7	*	0.7	ns	1.6	ns	3.1	**	4.7	*	1.6	ns	2.0	***	0.9	ns
Error	11.5		56.6		2.3		5.8		8.9		8.5		15.2		8.7		2.1		5.6	
Means																				
Maturity	(mg g^−1^ dw)		(mg g^−1^ dw)		(mg g^−1^ dw)		(mg g^−1^ dw)		(mg g^−1^ dw)		(mg g^−1^ dw)		(mg g^−1^ dw)		(mg g^−1^ dw)		(Ratio)		(Ratio)	
RS1	81.4 ± 3.8 b		95.4 ± 3.0		56.6 ± 5.8 d		233.4 ± 10.8 d		12.7 ± 0.6 a		2.5 ± 0.2 a		0.8 ± 0.1 a		16.0 ± 0.7 a		3.5 ± 0.3 a		15.1 ± 1.2 e	
RS2	71.9 ± 2.0 b		98.8 ± 2.1		64.3 ± 3.2 d		235.0 ± 5.3 d		13.1 ± 0.6 a		2.7 ± 0.2 a		0.6 ± 0.0 b		16.4 ± 0.8 a		2.7 ± 0.2 b		14.7 ± 0.8 e	
RS3	64.1 ± 2.3 c		93.8 ± 3.7		176.9 ± 5.6 c		334.8 ± 9.4 c		10.9 ± 0.5 b		1.8 ± 0.1 b		0.6 ± 0.1 b		13.3 ± 0.6 b		0.9 ± 0.0 c		25.7 ± 1.1 d	
RS4	48.2 ± 1.7 d		89.4 ± 3.0		310.4 ± 10.3 b		448.0 ± 12.9 b		7.6 ± 0.4 c		1.4 ± 0.1 c		0.4 ± 0.1 c		9.4 ± 0.5 c		0.5 ± 0.0 d		49.3 ± 3.1 c	
RS5	44.7 ± 1.4 d		91.8 ± 3.2		327.6 ± 10.2 b		464.1 ± 10.0 b		7.0 ± 0.6 c		1.5 ± 0.1 c		0.3 ± 0.0 d		8.7 ± 0.7 c		0.4 ± 0.0 d		57.1 ± 4.8 b	
RS6	50.2 ± 1.7 d		95.2 ± 2.9		353.2 ± 7.6 a		498.7 ± 8.4 a		6.3 ± 0.5 c		1.3 ± 0.1 c		0.3 ± 0.0 d		7.8 ± 0.5 c		0.4 ± 0.0 d		67.3 ± 5.2 a	
Location																				
Kalavasos (15 m)	59.7 ± 2.2		96.1 ± 1.6		216.2 ± 20.8		371.9 ± 19.0		8.6 ± 0.6 b		1.9 ± 0.2 a		0.4 ± 0.0 b		10.9 ± 0.7 b		1.3 ± 0.2 b		43.9 ± 4.5 a	
Vavla (510 m)	60.5 ± 2.9		92.0 ± 1.9		213.6 ± 21.5		366.1 ± 19.0		10.6 ± 0.5 a		1.8 ± 0.1 b		0.6 ± 0.0 a		13.0 ± 0.6 a		1.5 ± 0.3 a		32.5 ± 2.9 b	
Genotype																				
Lefkaritiki	58.5 ± 1.8 b		95.8 ± 1.8		206.3 ± 21.0 b		360.5 ± 20.1 b		10.4 ± 0.6 a		2.1 ± 0.1 a		0.5 ± 0.0		13.0 ± 0.7 a		1.5 ± 0.3 a		34.2 ± 3.5 b	
Mavroteratsia	61.7 ± 3.2 a		92.3 ± 1.7		223.5 ± 21.2 a		377.5 ± 17.8 a		8.8 ± 0.5 b		1.6 ± 0.1 b		0.5 ± 0.0		10.9 ± 0.6 b		1.3 ± 0.2 b		42.2 ± 4.2 a	

Data represent three replicates, each consisting of six fruits, for each treatment. ns = nonsignificant effect; significant effect at the *p* ≤ 0.05 (*), *p* ≤ 0.01 (**) and *p* ≤ 0.001 (***) levels. Different letters within each row per effect indicate significant differences according to Tukey’s b test (*p* < 0.05).

**Table 4:** Identified compounds, retention time and exact mass spectra data for carob fruit polyphenols analyzed by UHPLC-Q-Orbitrap HRMS at the six critical stages of maturity, from fully developed green (RS1) to late ripe (RS6) fruit.

Compound	Theoretical Mass	Measured Mass	Accuracy (D ppm)	Tr (Min)
	m/z (M-H)^−^			
gallic acid	169.10143	169.101527	0.603	2.53
methyl gallate	183.02990	183.029770	−0.710	6.72
catechin	289.07176	289.071790	0.104	7.23
naringenin diglucoside	595.16684	595.166900	0.101	7.75
epicatechin	289.07176	289.071130	−2.179	7.94
epigallocatechin gallate	457.07763	457.077110	−1.138	7.93
gallocatechin gallate	457.07763	457.077130	−1.094	8.36
vitexin	431.09837	431.098130	−0.557	9.07
luteolin-7-*O*-glucoside	447.09328	447.094100	1.834	9.44
kaempferol-7-*O*-glucoside	447.09328	447.093980	1.566	10.05
isovitexin	431.09837	431.098860	1.137	10.55
gallocatechin	305.06668	305.065000	−5.507	10.87
epigallocatechin	305.06668	305.066790	0.361	7.44
quercetin 3-*O*-glucoside	463.08820	463.088340	0.302	9.6
quercetin3-ramnoside	447.09328	447.093560	0.626	10.22
tetragalloyl glucose	787.09994	787.098760	−1.499	10.22
trigalloyl glucose	635.08899	635.088760	−0.362	8.41
digalloyl glucose	483.07803	483.078650	1.283	7.66

**Table 5:** Analysis of variance, percentage of total variance and means ± SE for the concentrations of 18 polyphenols analyzed by UHPLC-Q-Orbitrap HRMS in the pulp of carob fruits derived from two locations (altitudes) and two genotypes at the six critical stages of maturity.

Source of Variance	Gallocatechin		Epigallocatechin gallate		Catechin		Gallocatechin gallate		Epigallocatechin		Epicatechin		Catechins		Naringenin diglucoside		Luteolin 7-O-glucoside		Vitexin		Isovitexin		Flavone glycosides		Kaempferol 3-O-glucoside		Quercetin 3-O-glucoside		Quercetin 3-ramnoside		Flavonol glycosides		Tetragalloyl glucose		Trigalloyl glucose		Digalloyl glucose		Hydrolyzable tannins		Gallic acid		Methyl gallate	
	PTV	Sig	PTV	Sig	PTV	Sig	PTV	Sig	PTV	Sig	PTV	Sig	PTV	Sig	PTV	Sig	PTV	Sig	PTV	Sig	PTV	Sig	PTV	Sig	PTV	Sig	PTV	Sig	PTV	Sig	PTV	Sig	PTV	Sig	PTV	Sig	PTV	Sig	PTV	Sig	PTV	Sig	PTV	Sig
Maturity (M)	59.3	***	87.9	***	70.3	***	87.4	***	75.4	***	86.3	***	83.7	***	14.6	***	34.2	***	42.9	***	50.2	***	4.8	***	59.0	***	49.2	***	55.9	***	56.6	***	85.4	***	68.4	***	49.6	***	83.2	***	78.8	***	78.5	***
Location (L)	1.0	***	1.4	***	6.5	***	0.3	**	3.6	***	3.4	***	0.2	***	0.1		3.9	***	0.4	**	0.0		1.6	**	0.2		0.7	***	0.0		0.1		1.4	***	5.4	***	8.6	***	2.8	***	6.3	***	5.0	***
Genotype (G)	1.0	***	0.0		4.4	***	0.3	**	2.4	***	0.1	*	0.8	***	0.4		0.6	*	0.1		4.5	***	2.7	***	16.5	***	9.9	***	17.9	***	15.8	***	0.1		2.0	***	0.2		0.3	**	0.2	**	0.4	***
M × L	15.1	***	5.0	***	6.3	***	5.2	***	3.4	***	8.7	***	3.9	***	40.1	***	19.4	***	8.6	***	29.9	***	50.0	***	4.1	***	9.1	***	6.6	***	5.5	***	3.4	***	10.4	***	9.4	***	3.0	***	7.0	***	9.1	***
M × G	7.7	***	1.1	***	10.8	***	0.8	**	8.7	***	0.2	**	3.5	***	16.8	***	20.8	***	14.5	***	3.8	***	23.3	***	6.9	***	11.5	***	5.5	***	6.6	***	0.8	**	1.4	***	8.3	***	0.8	***	3.9	***	4.5	***
L × G	6.9	***	2.1	***	0.1		2.0	***	0.0		0.1	**	3.6	***	4.0	***	4.2	***	11.3	***	3.3	***	0.9	**	8.3	***	8.4	***	8.0	***	9.5	***	2.7	***	5.4	***	5.7	***	3.8	***	0.1	*	0.6	***
M × L × G	8.6	***	1.9	***	1.0	***	2.7	***	5.9	***	0.5	***	4.0	***	17.5	***	11.4	***	19.5	***	5.1	***	11.9	***	2.6	***	10.4	***	3.5	***	4.8	***	5.2	***	5.7	***	12.1	***	5.2	***	3.0	***	1.0	***
Error	0.4		0.7		0.8		1.4		0.5		0.6		0.3		6.6		5.7		2.7		3.3		4.9		2.5		0.8		2.6		1.2		1.1		1.3		6.1		0.9		0.7		1.0	
Maturity	Means (μg g^−1^ dw)																																											
RS1	1070 ± 198 a		554 ± 56 b		600 ± 82 a		312 ± 37 b		9.9 ± 0.6 c		3.9 ± 0.2 c		2551 ± 239 a		2.5 ± 0.5 c		3.1 ± 0.6 b		1.8 ± 0.5 c		3.4 ± 0.5 a		10.7 ± 1.8 bc		80.4 ± 4.6 a		57.2 ± 4.1 b		52.7 ± 4.6 a		190.3 ± 11.8 a		575.7 ± 19.0 a		204.7 ± 21.9 a		85.8 ± 5.1 a		866.2 ± 39.4 a		0.0001 c		0.0001 d	
RS2	963 ± 143 b		594 ± 18 a		371 ± 39 c		557 ± 31 a		18.0 ± 2.4 a		10.9 ± 1.1 a		2514 ± 162 a		3.4 ± 0.3 b		2.7 ± 0.2 b		2.5 ± 0.6 b		2.9 ± 0.3 b		11.5 ± 0.7 ab		59.8 ± 5.6 c		80.0 ± 16.1 a		42.0 ± 4.3 b		181.8 ± 25.1 a		402.1 ± 20.1 b		207.8 ± 9.2 a		84.8 ± 3.6 a		694.6 ± 29.8 b		0.0001 c		0.0001 d	
RS3	565 ± 129 c		318 ± 23 c		414 ± 61 b		219 ± 31 c		13.7 ± 1.7 b		6.8 ± 0.3 b		1537 ± 213 b		4.3 ± 0.4 a		5.0 ± 0.5 a		0.3 ± 0.1 d		2.3 ± 0.3 c		11.8 ± 0.6 a		67.2 ± 9.8 b		57.4 ± 8.7 b		34.8 ± 5.3 c		159.4 ± 22.4 b		414.4 ± 47.1 b		144.6 ± 13.8 b		58.6 ± 2.3 b		617.6 ± 61.1 c		0.0001 c		0.0001 d	
RS4	32 ± 4 d		57 ± 6 d		39 ± 4 d		11 ± 2 d		0.6 ± 0.1 d		0.5 ± 0.1 d		140 ± 11 c		3.2 ± 0.3 b		2.7 ± 0.1 b		3.0 ± 0.3 a		1.5 ± 0.1 d		10.4 ± 06 c		35.3 ± 3.6 d		15.5 ± 2.5 c		25.6 ± 2.5 d		76.4 ± 7.2 c		106.3 ± 10.4 cd		77.1 ± 6.1 c		62.4 ± 4.6 b		245.8 ± 19.9 d		115.9 ± 14.8 b		0.44 ± 0.06 c	
RS5	7 ± 1 d		55 ± 4 d		32 ± 4 d		5 ± 1 d		0.3 ± 0.1d		0.2 ± 0.0 d		100 ± 8 c		3.3 ± 0.4 b		2.8 ± 0.2 b		3.2 ± 0.2 a		1.2 ± 0.2 e		10.5 ± 0.7 c		22.9 ± 5.0 e		17.0 ± 3.2 c		14.0 ± 3.4 e		53.8 ± 11.5 d		122.5 ± 12.9 c		81.8 ± 8.5 c		56.9 ± 4.6 b		261.1 ± 22.2 d		121.0 ± 17.3 b		0.59 ± 0.07 a	
RS6	7 ± 1 d		33 ± 6 d		17 ± 4 d		2 ± 0 d		0.2 ± 0.0 d		0.2 ± 0.0 d		64 ± 12 c		3.6 ± 0.3 b		2.8 ± 0.1 b		2.3 ± 0.1 b		1.1 ± 0.1 e		9.8 ± 0.3 c		22.5 ± 2.0 e		14.8 ± 2.2 c		14.6 ± 0.7 e		51.9 ± 4.7 d		91.0 ± 5.7 d		63.0 ± 6.0 d		47.5 ± 5.3 c		201.5 ± 13.5 e		215.5 ± 21.7 a		0.51 ± 0.03 b	
Location																																												
Kalavasos (15 m)	500 ± 121 a		240 ± 38 b		176 ± 35 b		172 ± 38 b		5.5 ± 1.1 b		2.9 ± 0.5 b		1097 ± 217 b		3.3 ± 0.3		2.9 ± 0.1 b		2.1 ± 0.2 b		2.1 ± 0.2		10.4 ± 0.5 b		46.9 ± 5.2		43.5 ± 7.8 a		30.9 ± 3.3		121.2 ± 15.5		261.1 ± 28.7 b		113.1 ± 9.0 b		60.0 ± 3.9 b		434.2 ± 39.0 b		98.6 ± 18.9 a		0.32 ± 0.06 b	
Vavla (510 m)	382 ± 69 b		299 ± 46 a		315 ± 52 a		196 ± 36 a		8.7 ± 1.6 a		4.5 ± 0.9 a		1205 ± 189 a		3.4 ± 0.2		3.5 ± 0.3 a		2.3 ± 0.3 a		2.0 ± 0.2		11.2 ± 0.6 a		49.2 ± 4.6		37.2 ± 3.9 b		30.4 ± 3.1		116.7 ± 10.9		309.5 ± 38.7 a		146.6 ± 14.2 a		72.0 ± 2.5 a		528.1 ± 53.5 a		52.2 ± 10.1 b		0.19 ± 0.04 a	
Genotype																																												
Lefkaritiki	381 ± 74 b		268 ± 44		189 ± 34 b		196 ± 40 a		5.8 ± 1.1 b		3.6 ± 0.7 b		1043 ± 180 b		3.3 ± 0.2		3.1 ± 0.2 b		2.1 ± 0.3		1.8 ± 0.2 b		10.3 ± 0.4 b		36.2 ± 4.0 b		28.8 ± 3.8 b		22.6 ± 2.3 b		87.6 ± 9.5 b		290.8 ± 36.4		139.9 ± 13.6 a		65.2 ± 3.5		495.9 ± 50.6 a		79.0 ± 14.8 a		0.24 ± 0.04 b	
Mavroteratsia	500 ± 119 a		271 ± 41		302 ± 54 a		172 ± 33 b		8.4 ± 1.6 a		3.8 ± 0.7 a		1259 ± 223 a		3.5 ± 0.2		3.3 ± 0.3 a		2.2 ± 0.2		2.3 ± 0.2 a		11.3 ± 0.6 a		59.9 ± 4.9 a		51.9 ± 7.4 a		38.6 ± 3.4 a		150.3 ± 14.5 a		279.9 ± 32.0		119.7 ± 10.5 b		66.8 ± 3.4		466.4 ± 44.1 b		71.8 ± 16.4 b		0.28 ± 0.05 a	

Data represent three replicates, each consisting of six fruits, for each treatment. ns = nonsignificant effect; significant effect at the *p* ≤ 0.05 (*), *p* ≤ 0.01 (**) and *p* ≤ 0.001 (***) levels. Different letters within each row per effect indicate significant differences according to Tukey’s b test (*p* < 0.05).

**Table 6:** Analysis of variance, percent of total variance and means ± SE for the total phenolic content, condensed tannins content, ferric-reducing antioxidant power (FRAP) and 1,1-diphenyl-2-picrylhydrazyl free-radical (DPPH) scavenging capacity in the pulp of carob fruits derived from two locations (altitudes) and two genotypes at the six critical stages of maturity.

Source of Variance	Total Phenolics		Condensed Tannins		FRAP		DPPH	
	PTV	Sig	PTV	Sig	PTV	Sig	PTV	Sig
Maturity (M)	98.0	***	93.8	***	95.8	***	97.7	***
Location (L)	0.2	**	2.1	***	1.0	***	0.1	**
Genotype (G)	0.1	**	0.3	***	0.3	***	0.0	ns
M × L	0.3	**	2.8	***	1.5	***	0.2	ns
M × G	0.5	***	0.3	***	0.2	*	0.1	ns
L × G	0.0	ns	0.1	***	0.0	ns	0.3	***
M × L × G	0.1	ns	0.4	***	0.6	***	0.9	***
Error	0.7		0.2		0.7		0.7	
Means								
Maturity	(mg g^−1^ dw)		(mg g^−1^ dw)		(μmol AAE g^−1^ dw)		(μmol AAE g^−1^ dw)	
RS1	181.1 ± 3.7 a		198.3 ± 2.9 a		1030 ± 11 a		1712 ± 44 a	
RS2	172.9 ± 4.1 b		196.8 ± 3.9 a		989 ± 16 b		1500 ± 56 b	
RS3	116.9 ± 3.5 c		124.8 ± 3.2 b		735 ± 11 c		789 ± 12 c	
RS4	30.7 ± 2.8 d		52.0 ± 3.2 c		409 ± 15 d		190 ± 21 d	
RS5	17.4 ± 2.0 e		54.8 ± 6.3 c		354 ± 28 e		76 ± 10 e	
RS6	17.0 ± 1.3 e		46.5 ± 8.5 d		324 ± 25 f		73.5 ± 7 e	
Location								
Kalavasos (15 m)	86.1 ± 12.5 b		102.4 ± 12.5 b		610 ± 55 b		702 ± 118 b	
Vavla (510 m)	92.5 ± 11.6 a		122.0 ± 10.1 a		670 ± 45 a		745 ± 111 a	
Genotype								
Lefkaritiki	91.8 ± 12.4 a		116.0 ± 11.4 a		657 ± 51 a		736 ± 116	
Mavroteratsia	86.8 ± 11.7 b		108.4 ± 11.6 b		624 ± 50 b		711 ± 114	

Data represent three replicates, each consisting of six fruits, for each treatment. ns = nonsignificant effect; significant effect at the *p* ≤ 0.05 (*), *p* ≤ 0.01 (**) and *p* ≤ 0.001 (***) levels. Different letters within each row per effect indicate significant differences according to Tukey’s b test (*p* < 0.05).

## Data Availability

The data presented in this study are available on request from the corresponding author. The data are not publicly available due to reasons of privacy.

## Supplementary Materials

The following are available online at https://www.mdpi.com/2076-3921/10/1/57/s1, Table S1: Mean daily maximum and minimum temperature.

## References

[1] Tous J., Romero A., Batlle I. (2013). The Carob tree: Botany, horticulture, and genetic resources. Hortic. Rev..

[2] Goulas V., Stylos E., Chatziathanasiadou M.V., Mavromoustakos T., Tzakos A.G. (2016). Functional Components of Carob Fruit: Linking the Chemical and Biological Space. Int. J. Mol. Sci..

[3] Batlle I., Tous J. (1997). Promoting the conservation and use of underutilized and neglected crops. Carob Tree: Ceratonia siliqua L..

[4] Bouzouita N., Khaldi A., Zgoulli S., Chebil L., Chekki R., Chaabouni M., Thonart P. (2007). The analysis of crude and purified locust bean gum: A comparison of samples from different carob tree populations in Tunisia. Food Chem..

[5] Avallone R., Plessi M., Baraldi M., Monzani A. (1997). Determination of Chemical Composition of Carob (Ceratonia siliqua): Protein, Fat, Carbohydrates, and Tannins. J. Food Compos. Anal..

[6] Stavrou I.J., Christou A., Kapnissi-Christodoulou C.P. (2018). Polyphenols in carobs: A review on their composition, antioxidant capacity and cytotoxic effects, and health impact. Food Chem..

[7] Zunft H., Lüder W., Harde A., Haber B., Graubaum H.J., Koebnick C., Grünwald J. (2003). Carob pulp preparation rich in insoluble fibre lowers total and LDL cholesterol in hypercholesterolemic patients. Eur. J. Nutr..

[8] Theophilou I.C., Neophytou C.M., Constantinou A.I. (2017). Carob and its Components in the Management of Gastrointestinal Disorders. J. Hepatol. Gastroenterol..

[9] Nasar-Abbas S.M., Huma Z., Vu T.-H., Khan M.K., Esbenshade H., Jayasena V. (2016). Carob Kibble: A Bioactive-Rich Food Ingredient. Compr. Rev. Food Sci. Food Saf..

[10] Yousif A.K., Alghzawi H. (2000). Processing and characterization of carob powder. Food Chem..

[11] Bosch J., Del Pino F.G., Ramoneda J., Retana J. (1996). Fruiting phenology and fruit set of carob, Ceratonia siliqua L.(Cesalpinaceae). Isr. J. Plant Sci..

[12] Von Haselberg C.D. (2000). Vegetative Growth and Flower and Fruit Development in Carob Trees (Ceratonia siliqua L.) with Special Emphasis on Environmental Conditions at Marginal Production Sites in South Portugal.

[13] Morton J.F., Julia F. (1987). Fruits of Warm Climates.

[14] Benchikh Y., Louaileche H., George B., Merlin A. (2014). Changes in bioactive phytochemical content and in vitro antioxidant activity of carob (*Ceratonia siliqua* L.) as influenced by fruit ripening. Ind. Crops Prod..

[15] Farag M.A., El-Kersh D.M., Ehrlich A., Choucry M.A., El-Seedi H., Frolov A., Wessjohann L.A. (2019). Variation in Ceratonia siliqua pod metabolome in context of its different geographical origin, ripening stage and roasting process. Food Chem..

[16] Ydjedd S., Chaala M., Richard G., Kati D.E., López-Nicolás R., Fauconnier M.-L., Louaileche H. (2017). Assessment of antioxidant potential of phenolic compounds fractions of Algerian Ceratonia siliqua L. pods during ripening stages. Int. Food Res. J..

[17] Vekiari A., Ouzounidou G., Gork G., Ozturk M., Asfi M. (2012). Compositional changes of major chemical compounds in Greek carob pods during development. Bull. Chem. Soc. Ethiop..

[18] Othmen K.B., Elfalleh W., Lachiheb B., Haddad M. (2019). Evolution of phytochemical and antioxidant activity of Tunisian carob (*Ceratonia siliqua* L.) pods during maturation. EuroBiotech J..

[19] Kyratzis A., Antoniou C., Papayiannis L.C., Graziani G., Rouphael Y., Kyriacou M.C. (2020). Pod morphology, primary and secondary metabolite profiles in non-grafted and grafted carob germplasm are configured by agro-environmental zone, genotype and growing season. Front. Plant Sci..

[20] Kyriacou M.C., Emmanouilidou M.G., Soteriou G.A. (2016). Asynchronous ripening behavior of cactus pear (Opuntia ficus-indica) cultivars with respect to physicochemical and physiological attributes. Food Chem..

[21] Rouphael Y., Colla G., Giordano M., El-Nakhel C., Kyriacou M.C., De Pascale S. (2017). Foliar applications of a legume-derived protein hydrolysate elicit dose-dependent increases of growth, leaf mineral composition, yield and fruit quality in two greenhouse tomato cultivars. Sci. Hortic..

[22] Bremner J. (1965). Total nitrogen. Methods of Soil Analysis: Part 2 Chemical and Microbiological Properties, 9.2.

[23] Antoniou C., Kyratzis A., Rouphael Y., Stylianou S., Kyriacou M. (2020). Heat-and Ultrasound-Assisted Aqueous Extraction of Soluble Carbohydrates and Phenolics from Carob Kibbles of Variable Size and Source Material. Foods.

[24] Singleton V.L., Orthofer R., Lamuela-Raventós R.M. (1999). Analysis of total phenols and other oxidation substrates and antioxidants by means of folin-ciocalteu reagent. Methods in Enzymology.

[25] Sun B., Ricardo-da-Silva J.M., Spranger I. (1998). Critical Factors of Vanillin Assay for Catechins and Proanthocyanidins. J. Agric. Food Chem..

[26] Sepperer T., Hernandez-Ramos F., Labidi J., Oostingh G.J., Bogner B., Petutschnigg A., Tondi G. (2019). Purification of industrial tannin extract through simple solid-liquid extractions. Ind. Crop. Prod..

[27] Rached I., Barros L., Fernandes I.P., Santos-Buelga C., Rodrigues A.E., Ferchichi A., Barreiro M.F., Ferreira I.C. (2016). Ceratonia siliqua L. hydroethanolic extract obtained by ultrasonication: Antioxidant activity, phenolic compounds profile and effects in yogurts functionalized with their free and microencapsulated forms. Food Fun..

[28] Kyriacou M.C., Ioannidou S., Nikoloudakis N., Seraphides N., Papayiannis L.C., Kyratzis A.C. (2020). Physicochemical characterization and trait stability in a genetically diverse ex situ collection of pomegranate (*Punica granatum* L.) germplasm from Cyprus. Sci. Hortic..

[29] Brand-Williams W., Cuvelier M.-E., Berset C. (1995). Use of a free radical method to evaluate antioxidant activity. LWT-Food Sci. Technol..

[30] Benzie I.F.F., Strain J.J. (1996). The Ferric Reducing Ability of Plasma (FRAP) as a Measure of “Antioxidant Power”: The FRAP Assay. Anal. Biochem..

[31] McGuire R.G.J.H. (1992). Reporting of objective color measurements. HortScience.

[32] Rupasinghe H.V. (2008). The role of polyphenols in quality, postharvest handling, and processing of fruits. Postharvest Biology and Technology of Fruits, Vegetables and Flowers.

[33] Özcan M.M., Arslan D., Gökçalik H. (2007). Some compositional properties and mineral contents of carob (Ceratonia siliqua) fruit, flour and syrup. Int. J. Food Sci. Nutr..

[34] Kokkinofta R., Yiannopoulos S., Stylianou M.A., Agapiou A. (2020). Use of Chemometrics for Correlating Carobs Nutritional Compositional Values with Geographic Origin. Metabolites.

[35] Kader A.A. (2002). Postharvest Technology of Horticultural Crops.

[36] Valero D., Serrano M. (2010). Postharvest Biology and Technology for Preserving Fruit Quality.

[37] Levander O.A. (1990). Fruit and vegetable contributions to dietary mineral intake in human health and disease. HortScience.

[38] Gharibzahedi S.M.T., Jafari S.M. (2017). The importance of minerals in human nutrition: Bioavailability, food fortification, processing effects and nanoencapsulation. Trends Food Sci. Technol..

[39] United States Department of Agriculture Food Database.

[40] Simsek S., Ozcan M.M., Al Juhaimi F., ElBabiker E., Ghafoor K. (2017). Amino Acid and Sugar Contents of Wild and Cultivated Carob (*Ceratonia siliqua*) Pods Collected in Different Harvest Periods. Chem. Nat. Compd..

[41] Kyriacou M.C., Rouphael Y. (2018). Towards a new definition of quality for fresh fruits and vegetables. Sci. Hortic..

[42] Roseiro L.B., Duarte L.C., Oliveira D.L., Roque R., Bernardo-Gil M.G., Martins A.I., Sepúlveda C., Almeida J., Meireles M., Gírio F.M. (2013). Supercritical, ultrasound and conventional extracts from carob (*Ceratonia siliqua* L.) biomass: Effect on the phenolic profile and antiproliferative activity. Ind. Crops Prod..

[43] Alañón M.E., Oliver-Simancas R., Gómez-Caravaca A.M., Arráez-Román D., Segura-Carretero A. (2019). Evolution of bioactive compounds of three mango cultivars (*Mangifera indica* L.) at different maturation stages analyzed by HPLC-DAD-q-TOF-MS. Food Res. Int..

[44] Cheynier V., Comte G., Davies K.M., Lattanzio V., Martens S. (2013). Plant phenolics: Recent advances on their biosynthesis, genetics, and ecophysiology. Plant Physiol. Biochem..

[45] Crozier A., Jaganath I.B., Clifford M.N. (2006). Phenols, polyphenols and tannins: An overview. Plant Secondary Metabolites: Occurrence, Structure and Role in the Human Diet.

[46] Yang B., Liu P. (2014). Composition and Biological Activities of Hydrolyzable Tannins of Fruits of Phyllanthus emblica. J. Agric. Food Chem..

[47] Lestario L.N., Howard L.R., Brownmiller C., Stebbins N.B., Liyanage R., Lay J.O. (2017). Changes in polyphenolics during maturation of Java plum (Syzygium cumini Lam.). Food Res. Int..

[48] Vogt T. (2010). Phenylpropanoid Biosynthesis. Mol. Plant.

[49] Josuttis M., Verrall S., Stewart D., Krüger E., McDougall G.J. (2013). Genetic and Environmental Effects on Tannin Composition in Strawberry (Fragaria × ananassa) Cultivars Grown in Different European Locations. J. Agric. Food Chem..

[50] Rice-Evans C.A., Miller N.J., Paganga G. (1996). Structure-antioxidant activity relationships of flavonoids and phenolic acids. Free Radic. Biol. Med..

[51] Custodio L., Fernandes E., Escapa A.L., Fajardo A., Aligue R., Albericio F., Neng N.R., Nogueira J.M., Romano A. (2011). Antioxidant and cytotoxic activities of carob tree fruit pulps are strongly influenced by gender and cultivar. J. Agric. Food Chem..

[52] Sebai H., Souli A., Chehimi L., Rtibi K., Amri M., El-Benna J., Sakly M. (2013). In vitro and in vivo antioxidant properties of Tunisian carob (*Ceratonia siliqua* L.). J. Med. Plants Res..

[53] Kaneria M.J., Bapodara M.B., Chanda S.V. (2012). Effect of Extraction Techniques and Solvents on Antioxidant Activity of Pomegranate (*Punica granatum* L.) Leaf and Stem. Food Anal. Methods.

[54] Santos-Buelga C., Scalbert A. (2000). Proanthocyanidins and tannin-like compounds—Nature, occurrence, dietary intake and effects on nutrition and health. J. Sci. Food Agric..

[55] Gill S.S., Tuteja N. (2010). Reactive oxygen species and antioxidant machinery in abiotic stress tolerance in crop plants. Plant Physiol. Biochem..

[56] Kiss A.K., Piwowarski J.P. (2018). Ellagitannins, gallotannins and their metabolites-the contribution to the anti-inflammatory effect of food products and medicinal plants. Curr. Med. Chem..

[57] Bernatoniene J., Kopustinskiene D.M. (2018). The Role of Catechins in Cellular Responses to Oxidative Stress. Molecules.

[58] Lu Z., Nie G., Belton P.S., Tang H., Zhao B. (2006). Structure–activity relationship analysis of antioxidant ability and neuroprotective effect of gallic acid derivatives. Neurochem. Int..

