# Supplementary material for: Mapping the Primary and Secondary Metabolomes of Carob (Ceratonia siliqua L.) Fruit and Its Postharvest Antioxidant Potential at Critical Stages of Ripening
Source: Antioxidants (Basel). 2021 Jan 5;10(1):57. doi: 10.3390/antiox10010057 (PMC7824902; doi:10.3390/antiox10010057)
Supplement: Supplementary file 1 [file antioxidants-10-00057-s001.pdf]

Supplementary Table 1: Mean daily maximum and minimum temperature. precipitation and relative humidity on average records.

	Mean Daily Maximum Temperature (°C)		Mean Daily Minimum Temperature (°C)		Precipitation (mm)		Relative humidity (%)	
**Month**	**Vavla**	**Kalavasos**	**Vavla**	**Kalavasos**	**Vavla**	**Kalavasos**	**Vavla**	**Kalavasos**
**January**	15.35	17.25	4.97	6.23	96.50	86.50	76.17	80.00
**February**	16.85	18.07	5.32	6.85	72.60	59.50	73.50	78.00
**March**	20.07	20.30	6.88	7.58	53.30	39.20	65.83	72.00
**April**	24.22	23.62	9.85	10.23	30.30	15.30	57.17	62.00
**May**	29.67	27.62	13.93	14.12	28.10	7.70	50.50	60.00
**June**	33.53	30.63	17.57	17.48	16.00	1.70	49.00	61.00
**July**	37.02	33.05	20.53	19.88	5.00	0.70	43.00	63.00
**August**	36.83	33.28	20.25	20.52	3.50	0.20	48.50	65.00
**September**	34.15	31.67	17.58	18.33	11.80	3.50	51.17	62.00
**October**	29.03	28.73	14.40	15.72	36.30	26.50	57.67	60.00
**November**	22.80	23.90	10.35	12.13	51.60	52.30	67.17	69.00
**December**	17.77	19.35	7.05	8.68	106.60	101.50	76.83	79.00
**Mean**	26.44	25.62	12.39	13.15			59.71	67.58
**Total**					511.60	394.60		
